# The Risk of Arterial Thrombosis in Patients With Chronic Myeloid Leukemia Treated With Second and Third Generation BCR-ABL Tyrosine Kinase Inhibitors May Be Explained by Their Impact on Endothelial Cells: An In-Vitro Study

**DOI:** 10.3389/fphar.2020.01007

**Published:** 2020-07-03

**Authors:** Hélène Haguet, Céline Bouvy, Anne-Sophie Delvigne, Elise Modaffari, Adeline Wannez, Pierre Sonveaux, Jean-Michel Dogné, Jonathan Douxfils

**Affiliations:** ^1^ Department of Pharmacy, Namur Thrombosis and Hemostasis Center (NTHC), Namur Research Institute for Life Sciences (NARILIS), University of Namur, Namur, Belgium; ^2^ QUALIblood s.a., Namur, Belgium; ^3^ Pole of Pharmacology and Therapeutics, Institut de Recherche Expérimentale et Clinique (IREC), Université catholique de Louvain (UCLouvain), Brussels, Belgium

**Keywords:** BCR-ABL tyrosine kinase inhibitor, chronic myeloid leukemia, endothelial cells, atherosclerosis, cardiovascular

## Abstract

BCR-ABL tyrosine kinase inhibitors (TKIs) revolutionized the treatment of chronic myeloid leukemia, inducing deep molecular responses, largely improving patient survival and rendering treatment-free remission possible. However, three of the five BCR-ABL TKIs, dasatinib, nilotinib, and ponatinib, increase the risk of developing arterial thrombosis. Prior investigations reported that nilotinib and ponatinib affect the endothelium, but the mechanisms by which they exert their toxic effects are still unclear. The impact of dasatinib and bosutinib on endothelial cells has been poorly investigated. Here, we aimed to provide an *in vitro* homogenous evaluation of the effects of BCR-ABL TKIs on the endothelium, with a special focus on the type of cell death to elucidate the mechanisms responsible for the potential cytotoxic effects of BCR-ABL TKIs nilotinib and ponatinib on endothelial cells. We tested the five BCR-ABL TKIs at three concentrations on human umbilical venous endothelial cells (HUVECs). This study highlights the endothelial toxicity of ponatinib and provides insights about the mechanisms by which it affects endothelial cell viability. Ponatinib induced apoptosis and necrosis of HUVECs after 72 h. Dasatinib affected endothelial cells *in vitro* by inhibiting their proliferation and decreased wound closure as soon as 24 h of treatment and even at infra-therapeutic dose (0.005 µM). Comparatively, imatinib, nilotinib, and bosutinib had little impact on endothelial cells at therapeutic concentrations. They did not induce apoptosis nor necrosis, even after 72 h of treatment but they inhibited HUVEC proliferation. Overall, this study reports various effects of BCR-ABL TKIs on endothelial cells and suggests that ponatinib and dasatinib induce arterial thrombosis through endothelial dysfunction.

## Introduction

Since the approval in 2001 of imatinib, the first tyrosine kinase inhibitor (TKI) targeting BCR-ABL, the interest and use of BCR-ABL TKIs have continued to grow (Bhamidipati et al., 2013). The discovery of imatinib led to a revolution in the treatment of chronic myeloid leukemia (CML), providing strong and durable molecular responses in numerous patients and improving patient survival close to that of the general population (Iqbal and Iqbal, 2014). However, intolerance and resistance to imatinib arose, leading to the development and approval of dasatinib, nilotinib, and bosutinib, three second-generation BCR-ABL TKIs that are nevertheless less selective for BCR-ABL than imatinib (Gorre et al., 2001; Bixby and Talpaz, 2009). These three drugs were approved for second-line treatment at the time of their approval. However, because of their efficacy in inducing deeper molecular remission, an essential criterion for treatment cessation (*i.e.*, *BCR-ABL1* transcript below 0.01% for at least 2 years), they have been approved for the treatment of chronic-phase CML in first line (Brummendorf et al., 2015; Cortes et al., 2016; Hochhaus et al., 2016; National Comprehensive Cancer Network, 2019). Ponatinib was developed as a fifth BCR-ABL TKI (Cortes et al., 2012). Because of its efficacy against T315I-mutated BCR-ABL, a mutation that confers resistance to all other BCR-ABL TKIs (Cortes et al., 2012), this third generation drug is used to treat CML resistant to first line treatments. Altogether, the five drugs are the mainstay of CML treatment (National Comprehensive Cancer Network, 2019).

Although these treatments are highly effective for CML in chronic phase, three of them (dasatinib, nilotinib, and ponatinib) increase the risk of adverse vascular events, particularly the risk of arterial thrombosis associated to myocardial infarction, stroke, and peripheral artery disease (Cortes et al., 2015; Pasvolsky et al., 2015; Douxfils et al., 2016; Haguet et al., 2017). However, each BCR-ABL TKI exhibits a different clinical vascular safety profile, suggesting different mechanisms of pathogenesis. The most common adverse vascular events associated to dasatinib are pleural effusion and pulmonary arterial hypertension (Cortes et al., 2017), whereas arterial thrombosis occurs less frequently (Saglio et al., 2017; Valent et al., 2017). Nilotinib causes hyperglycemia and hypercholesterolemia in addition to arterial thrombosis (Racil et al., 2013; Rea et al., 2014), whereas ponatinib is the TKI associated with the highest arterial thrombotic risk (Haguet et al., 2018). Interestingly, the time-to-event (*i.e*., the time between the initiation of the treatment and the occurrence of arterial thrombosis) differs between molecules. Ponatinib is associated with a rapid development of vascular events (median time-to-event: 8.5–14.1 months), compared to nilotinib (median time-to-event: 19.0–47.0 months), whereas little is known concerning arterial thrombosis induced by dasatinib (Gagnieu et al., 2013; Cortes et al., 2015; Fossard et al., 2016; Minson et al., 2019). Even if adverse vascular events occur more frequently in patients with prior cardiovascular risk factors, they also affect patients with no attributable cardiovascular risk factors (Aichberger et al., 2011; Tefferi and Letendre, 2011). Conversely, it has been proposed that imatinib prevents the occurrence of such events (Kadowaki and Kubota, 2004), whereas bosutinib apparently presents a safer vascular profile than the other second generation TKIs (Valent et al., 2017).

Because of the wide implementation of second generation TKIs for chronic patient treatment, their long-term safety is of particular interest. Understanding the pathophysiology of adverse vascular events induced by dasatinib, nilotinib, and ponatinib could help to define suitable risk minimization measures and help in the selection of the first-line treatment for CML patients. To date, the mechanisms underlying vascular adverse events are not fully characterized. Several hypotheses have been set forward based on the clinical characteristics of the vascular events. In particular, the predominance of arterial events raised concerns about the impact of BCR-ABL TKIs on platelet functions, endothelial cells, and atherosclerosis, and excluded prothrombotic states to be responsible for these events (Fossard et al., 2016). However, prior investigation indicated that BCR-ABL TKIs have no or anti-aggregation effects on platelets, suggesting a low probability of the implication of platelets in the occurrence of vascular thrombosis (Haguet et al., 2018). Therefore, researches focused on endothelial cells and atherosclerosis.

Previous mechanistic studies interrogating the effect of BCR-ABL TKIs on the vasculature demonstrated that some of these treatments affect the endothelium. For ponatinib and nilotinib, an increasing amount of evidence indicates that their vascular toxicity is a consequence, at least partially, of their effect on endothelial cells. *In vitro*, they both decreased endothelial cell viability and promoted the expression of molecular patterns related to apoptosis and angiogenesis (Hadzijusufovic et al., 2017; Gover-Proaktor et al., 2018). For these reasons, it has been hypothesized that vascular endothelial cells could be at the very origin of vasculopathies induced by ponatinib and nilotinib, which would be responsible for direct endothelial dysfunction (Hadzijusufovic et al., 2017). Dasatinib has been tested essentially on pulmonary endothelial cells in order to elucidate the mechanism behind dasatinib-induced pulmonary hypertension. It induced apoptosis and increased reactive oxygen species (ROS) reactive oxygen species production by pulmonary endothelial cells (Guignabert et al., 2016). Comparatively, the viability of dasatinib-treated endothelial cells other than pulmonary was unchanged (Gover-Proaktor et al., 2018). It has already been hypothesized that the vascular toxicity of BCR-ABL TKI is due to their lack of specificity toward BCR-ABL. Indeed, all BCR-ABL TKIs also inhibit numerous kinases (Rix et al., 2007; Talbert et al., 2015).

There is a lack of homogeneity in the *in vitro* studies performed to investigate the effects of nilotinib and ponatinib on endothelial cells. Overall, the methods, the tested concentrations and the cell line used often differed, making arduous the comparison between studies and resulting in conflicting reports (Vrekoussis et al., 2006; Venalis et al., 2009; Katgi et al., 2015). Here, we aimed to provide a homogenous evaluation and comparison of the viability of endothelial cells exposed to each of the five commercially available BCR-ABL TKIs. Major endothelial functions, such as ROS generation, the expression of adhesion molecules, and migration, have also been assessed, given their implication in vascular homeostasis and atherosclerosis. A particular consideration has been paid to results with dasatinib and bosutinib, as their impact on endothelial cells had previously been barely investigated, to the exception of dasatinib on pulmonary endothelial cells. In addition, even if the impact of nilotinib and ponatinib on endothelial cells is now well considered, there is still shadow on the mechanism(s) by which they exert their cytotoxic effects. To contribute to a better knowledge of the pathophysiology, our study further aimed to clarify the mechanism(s) by which BCR-ABL TKIs impair endothelial cell survival *in vitro*.

## Materials and Methods

### Cell Culture

Human umbilical vein endothelial cells (HUVECs) were purchased from Lonza (Verviers, Belgium). Cells were cultured in endothelial cell growth basal medium (Lonza, CC-3121, Verviers, Belgium) supplemented with SingleQuots™ (Lonza, CC-4133, Verviers, Belgium) and maintained at 37°C in a humidified atmosphere containing 5% CO_2_. HUVECs were used at passages 2–5.

### Drugs

Imatinib mesylate, dasatinib, nilotinib, bosutinib, and ponatinib were from Absource Diagnostic (Munich, Germany). All experiments were carried out using clinically relevant TKI concentrations (50 nM, 500 nM, and 5 µM for imatinib, 5 nM, 50 nM, and 500 nM for dasatinib and ponatinib, and 20 nM, 200 nM, and 2 µM for nilotinib and bosutinib). These concentrations were selected to consider the binding of TKIs to plasma proteins. Thus, these concentrations reflect the free concentration of TKIs in patients taking 400 mg OD of imatinib, 100 mg OD of dasatinib, 400 mg BID of nilotinib, 500 mg OD of bosutinib, and 45 mg OD of ponatinib (**Table 1**) (Rivera et al., 2014).

**Table 1:** Summary of the findings of the impact of BCR-ABL TKIs on endothelial cells *in vitro*.

		Imatinib	Dasatinib	Nilotinib	Bosutinib	Ponatinib
	Clinically effective C_max_^*^	0.682 µM	0.072 µM	0.171 µM	0.209 µM	0.040 µM
**Assay**	**Measured parameters**					
**Endothelial cells exposed to BCR-ABL TKIs for 24 h**						
**MTS assay**	Mitochondrial activity	=	=	=	=	↘ (0.5 µM)
**LDH assay**	LDH release (membrane integrity)	↘ (0.05 µM)	=	=	=	↗ (0.05 µM)
**Apoptosis assay**	Early apoptosis	=	=	=	=	=
	Late apoptosis/necrosis	=	=	=	=	=
**Proliferation assay**	Cells in S-phase	↗ (0.05 µM) ↘ (5 µM)	↘↘ (0.05 µM) ↘↘↘ (0.5 µM)	↘ (2 µM)	↘↘↘ (2 µM)	=
**Adhesion molecule expression (ELISA)**	ICAM-1	=	↘ (0.5 µM)	↘ (2 µM)	↘ (0.02 µM) ↗ (2 µM)	↘ (0.005 µM; 0.05 µM) ↘↘↘ (0.5 µM)
	VCAM-1	↘ (0.5 µM)	↘ (0.5 µM)	↘ (2 µM)	=	↘ (0.005 µM; 0.05 µM) ↘↘↘↘ (0.5 µM)
	E-/P-selectin	=	↘ (0.5 µM)	=	=	↘ (0.5 µM)
**Endothelial cells exposed to BCR-ABL TKIs for 72 h**						
**MTS assay**	Mitochondrial activity	↗ (5 µM)	↗ (0.005 µM; 0.5 µM)	=	=	↘↘ (0.5 µM)
**LDH assay**	LDH release (membrane integrity)	↗↗ (0.05 µM; 0.5 µM)	↗↗ (0.005 µM; 0.05 µM; 0.5 µM)	↗↗ (0.02 µM; 0.2 µM; 2 µM)	↗↗ (0.02 µM; 0.2 µM; 2 µM)	↗↗ (0.005 µM; 0.5 µM) ↗↗↗ (0.05 µM)
**Apoptosis assay**	Early apoptosis	=	↘↘ (0.005 µM; 0.05 µM; 0.5 µM)	=	↘↘ (2 µM)	↗↗↗↗ (0.5 µM)
	Late apoptosis/necrosis	=	=	=	=	↗↗↗ (0.05 µM)
**Proliferation assay**	Cells in S-phase	↘ (0.05 µM)	=	↘ (0.02 µM) ↘↘ (2 µM)	↘↘ (0.2 µM)	=
**ROS assay**	ROS levels	=	=	=	=	=

This table summarizes the results obtain in this study. Only statistically significant differences are reported. The number of arrows represents the magnitude of the following of difference: ↘ (from 0 to −24%), ↘↘ (from −25 to −49%), ↘↘↘ (from −50 to −74%), and ↘↘↘↘ (from −75 to −100%) and ↗ (from 0 to 24%), ↗↗ (from 25 to 49%), ↗↗↗ (from 50 to 74%), and ↗↗↗↗ (from 75 to 100%). The concentrations that significantly differed from the control are indicated in brackets.

*Clinically effective C_max_ represents the C_max_ corrected for the functional effects of protein binding (Rivera et al., 2014).

C_max_, maximum serum concentration.

All experiments were performed in 10% dialyzed FBS from Thermo Fischer Scientific (Waltham, MA, USA) in order to minimize interactions with serum-associated proteins, unless indicated otherwise.

### Viability

Cell metabolic integrity and viability were assayed using MTS (Promega, Fitchburg, MA, USA) and LDH (Roche, Basel, Switzerland) kits, respectively. In brief, HUVECs were seeded in 96-well plates at a density of 2,500 cells/cm^2^. Four days after plating, cells were treated with a TKI or 0.2% DMSO (control) diluted in medium containing 10% dialyzed FBS. Cell metabolic integrity and viability were assessed after 24 and 72 h of treatment using MTS and LDH assays according to manufacturers’ instructions. The absorbance at 490 nm was measured with a SpectraMax ID3 (Molecular Devices, San Jose, CA, USA).

### Apoptosis

To assess apoptosis induced by BCR-ABL TKIs, HUVECs were stained with Annexin V-FITC (staining of phosphatidylserine) and 7-AAD (DNA staining) in accordance with manufacturer’s instructions (Abcam, Cambridge, UK). Briefly, HUVECs were plated in 12-well plates and exposed to a TKI or 0.2% DMSO (control) for 24 or 72 h. Cells were then stained with Annexin V-FITC and 7-AAD in binding buffer. Stained HUVECs were analyzed on a FACS verse flow cytometer (BD Bioscience, Franklin Lakes, NJ, USA). Early apoptotic (*i.e.*, cells with externalized phosphatidylserine but with preserved membrane integrity) and late apoptotic/necrotic cells (*i.e.*, cells with disrupted plasma membrane integrity) were counted based on the relative number of Annexin V-FITC^+^/7-AAD^-^ and 7-AAD^+^ cells, respectively, using the BD FACSuite^®^ software.

### Proliferation

Cell proliferation was assessed using the Click-it 5-ethynyl-2’-deoxyuridine (EdU) flow kit (Thermo Fisher Scientific). In addition to the staining of EdU, a DNA stain was added (FxCycle Violet stain, Thermo Fisher Scientific). Aphidicolin at 10 µM was used as a positive control (Sigma-Aldrich, Saint-Louis, MO, USA). Briefly, HUVECs were plated in 12-well plates and exposed to a TKI, 0.2% DMSO (control), or aphidicolin. After 24 or 72 h of incubation, EdU was added for 2 h. Cells were then fixed, permeabilized, and the EdU detection mix was added for 30 min at room temperature. Finally, DNA was stained by incubating cells with FxCycle Violet stain for 30 min. HUVECs were then analyzed by flow cytometry (BD FACSverse), based on the fact that cells in S-phase are those that incorporate EdU (FITC^+^) and cells in G0/G1 phase and in G2/M phase do not incorporate EdU but are FxCycle^−^ and FxCycle^+^, respectively. Data were analyzed using the BD FACSuite^®^ software.

### Reactive Oxygen Species Concentrations

After the exposure of HUVECs to one of the five TKIs or to 0.2% DMSO (control) for 72 h, ROS content was quantified using CM-H2DCFDA (Invitrogen, Carlsbad, CA, USA), following manufacturer’s instructions. The oxidized fluorescent product retained inside cells was quantified using the FACSverse flow cytometer. Data were analyzed using the BD FACSuite^®^ software. SIN-1 hydrochloride (Sigma-Aldrich) was used a positive control.

### Endothelial Cell Migration

Endothelial cell migration was evaluated by a scratch assay that monitors the ability of cells to migrate in a wound. HUVECs were cultured in 24-well plates until confluence, and were then pre-treated with a given TKI or 0.2% DMSO (control) in medium containing 1% FBS (to minimize the contribution of cell proliferation on scratch closure) or 10% FBS during 24 h. The confluent monolayer was scratched with a pipette tip, and wound closure dynamics were tracked using an inverted Leica DMi1 microscope (Weltzar, Germany). Pictures were captured at baseline and after 2, 4, 6, and 24 h of incubation, and analyzed using the Leica Application Suite software version 4.7 to quantify the extent of cell migration in the cell-free scratch.

### On-Cell ELISA

The expression of intercellular adhesion molecule-1 (ICAM-1), vascular cell adhesion molecule-1 (VCAM-1), and E-/P-selectin was measured by on-cell enzyme-linked immunosorbent assay (ELISA). Briefly, HUVECs were cultured in 96-well plates and exposed to a TKI or 0.2% DMSO (control) for 24 h. Cells were then activated with TNF-α (R&D Systems, Minneapolis, MN, USA) at 10 ng/ml for 4 h. After treatment, cells were fixed with 0.025% glutaraldehyde for 10 min, and blocked with PBS-BSA 1% for 2 h. Fixed cells were incubated overnight with monoclonal antibodies targeting human ICAM-1 (recombinant human ICAM-1 clone #14C11, R&D systems), VCAM-1 (recombinant human VCAM-1 clone #HAE-2Z, R&D systems), and E-/P-selectin (CD62E/P, clone #BBIG-E6, R&D systems), and finally incubated with a secondary horseradish peroxidase (HRP)-conjugated antibody for 1 h (mouse IgG HRP-conjugated antibody, R&D systems). After incubation with 100 µl of the HRP substrate (50 µl of hydrogen peroxide and 50 µl of tetramethylbenzidine; R&D systems) for 20 min, optical density was measured at 450 nm with an Infinite^®^ M200 PRO (Tecan, Mechelen, Belgium). Results are expressed as the mean of the absorbance values relative to control.

### Statistical Analyses

Results are expressed as the means of the differences between the treated value and the control ± standard error of the mean (SEM). All experiments were repeated independently at least three times (N = 3). Comparison between different conditions was performed using the Wilcoxon signed rank test for experiments for which a standard distribution could not be defined. For flow cytometry analyses, a one-sample t-test was used. All statistical analyses were performed using the Prism 8 software (GraphPad Software Inc., San Diego, CA, United States). Data that were statistically significant in comparison to controls are indicated with * (p < 0.05), ** (p < 0.01) or *** (p < 0.001).

## Results

### Impact of BCR-ABL TKIs on Endothelial Cell Viability

**Figure 1:**
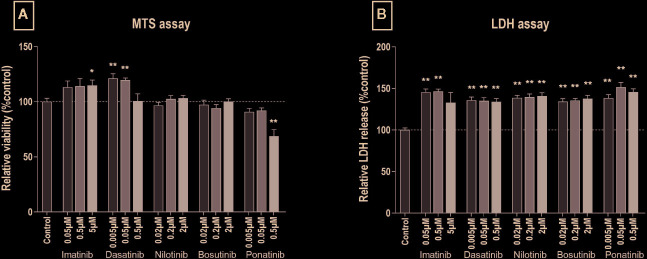
All BCR-ABL TKIs induce HUVEC membrane damage after 72 h. MTS **(A)** and LDH **(B)** assays were performed on HUVECs exposed for 72 h to the indicated BCR- ABL TKI in medium with 10% dialyzed FBS. Data are presented as means ± SEM of n = 9 of three independent experiments (N = 3). Results are expressed relative to control (DMSO 0.2%). Differences between conditions were tested using the Wilcoxon signed rank test that compared the effect of each TKI condition *versus* control. *p < 0.05 and **p < 0.01.

To test the cytotoxicity of BCR-ABL TKIs, HUVECs were exposed to three different concentrations of imatinib mesylate, dasatinib, nilotinib, bosutinib, or ponatinib for 24 or 72 h. Cell metabolic activity was tested using a MTS assay that reports on the activity of mitochondrial reductases (Buttke et al., 1993). Ponatinib exposure decreased cell metabolism after 24 and 72 h of treatment, whereas the other BCR-ABL TKIs did not (**Supplementary Figure 1A** and **Figure 1A**). Rather, HUVECs exposed to imatinib at 5 µM and dasatinib at 5 and 50 nM increased their metabolism. To link these results to cell viability, we performed a LDH assay that measures the leakage of LDH, an intracellular enzyme that is released from cells upon cell membrane damaged. After 24 h of treatment, TKIs did not induce more LDH release than control, to the exception of ponatinib at 0.05 µM that increased LDH release, suggesting that BCR-ABL TKIs do not induce early cell membrane damage (**Supplementary Figure 1B**). However, after 72 h of treatment, HUVEC membrane integrity was impacted, as shown by increased LDH release with all BCR-ABL TKIs (**Figure 1B**). Surprisingly, this alteration did not depend on the TKI concentration in the range that we tested.

**Figure 2:**
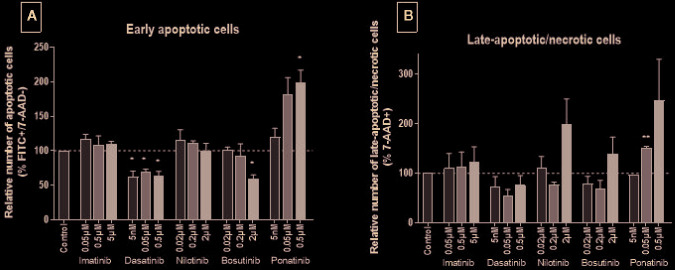
Ponatinib induces HUVEC apoptosis and necrosis. HUVECs were labeled with Annexin V^FITC^ and 7-AAD after exposure to BCR-ABL TKIs for 72 h in medium with 10% dialyzed FBS. The percentage of Annexin V^+^/7-AAD^−^ and 7-AAD^+^ cells revealed early apoptotic (*i.e.*, cells exposing phosphatidylserine but with preserved membrane integrity) **(A)** and late-apoptotic/necrotic (*i.e.*, cells with disrupted membrane integrity) **(B)** HUVECs, respectively. Bars represent the means of three experiments ± SEM. Results are expressed relative to control (DMSO 0.2%). Differences between conditions were tested using a one sample t-test that compared each TKI condition *versus* control. *p < 0.05 and **p < 0.01.

Because MTS and LDH assays do not fully discriminate the different cell death modes and to clarify the mechanism(s) by which BCR-ABL TKIs impair endothelial cell survival, we next tested apoptosis and late apoptosis/necrosis using specific FACS assays. After 24 h of incubation, TKIs did not induce apoptosis or late apoptosis/necrosis (**Supplementary Figure 2**). However, after 72 h, ponatinib dose-dependently induced HUVEC apoptosis and necrosis (**Figure 2**). Interestingly, dasatinib at all concentrations and bosutinib at 2 µM reduced the number of apoptotic cells. The other BCR-ABL TKIs did not significantly modulate HUVEC apoptosis nor necrosis.

**Figure 3:**
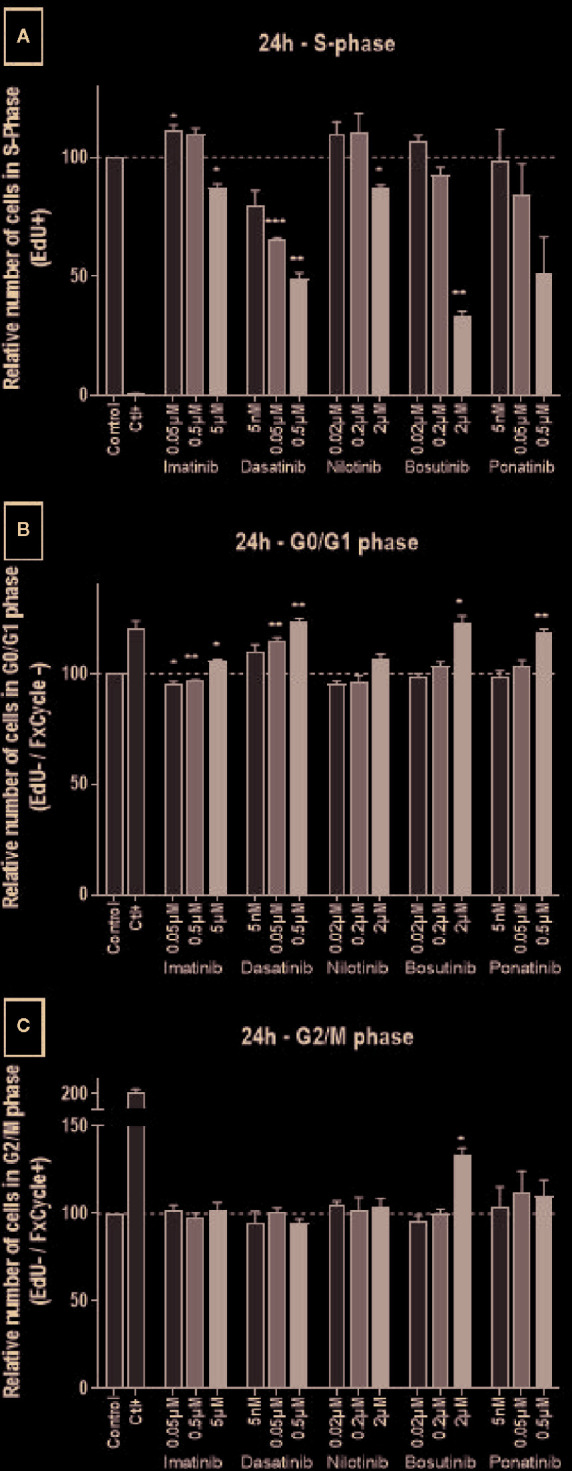
All BCR-ABL TKIs inhibit HUVEC proliferation. Cell cycle analysis was performed on HUVECs exposed to BCR-ABL TKIs for 24 h in medium with 10% dialyzed FBS by measuring EdU incorporation and DNA content (FxCycle). Histograms represent cells in S-phase **(A)**, G0/G1 phase **(B)**, and G2/M phase **(C)**. Bars represent the means of the three experiments ± SEM. Three concentrations were tested for each TKI. Results are expressed relative to control (DMSO 0.2%). Differences between conditions were tested using a one sample t-test that compared each TKI condition *versus* control. *p < 0.05 and **p < 0.01.

For what concerns cell cycling, all BCR-ABL TKIs influenced HUVECs as early as 24 h after treatment, as shown by a decreased of number of cells in the S-phase and an increased number of cells in the G0-G1 phase (**Figure 3**), suggesting that these drugs block HUVEC progression from G1 to S. Responses were dose-dependent, but they were seen only at the highest, supra-therapeutic concentrations of imatinib, nilotinib, and bosutinib. Bosutinib differed from the other TKIs in that, at the highest concentration, it also increased cells in the G2-M phase (**Figure 3**). Imatinib had the slightest effect on endothelial cell proliferation. After 72 h of treatment, inhibition of proliferation was still detected in HUVECs exposed to imatinib, nilotinib, and bosutinib (**Supplementary Figure 3**).

**Figure 4:**
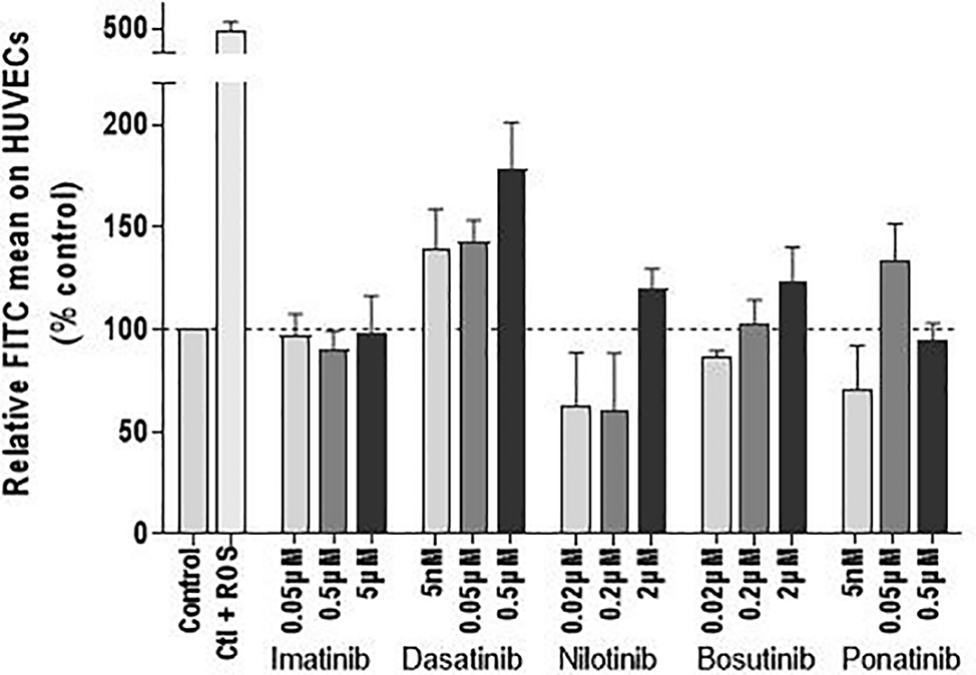
BCR-ABL TKIs do not increase the ROS levels in HUVECs. ROS levels in HUVECs after treatment with BCR-ABL TKIs for 72 h in medium with 10% dialyzed FBS, expressed as intensity of FITC. Bars represent the means of three experiments ± SEM. Results are expressed relative to control (DMSO 0.2%). Differences between conditions were tested using a one sample t-test that compared each TKI condition *versus* control.

To complete the investigation of the mechanism by which BCR-ABL TKIs impact endothelial cell viability, we evaluated their impact on ROS levels. ROS concentration in HUVECs was not significantly modified by BCR-ABL TKIs (**Figure 4**).

### Endothelial Cell Migration

**Figure 5:**
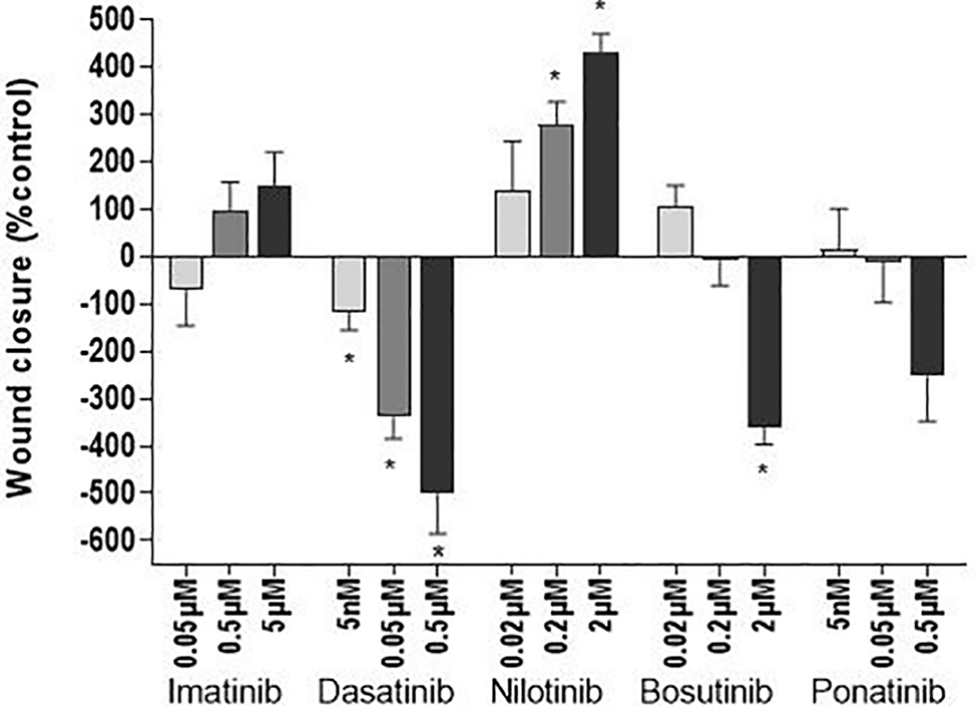
Dasatinib and bosutinib reduce wound closure. HUVEC migration was assessed by a scratch test after exposure of the cells to BCR-ABL TKIs for 24 h in 10% FBS media to avoid cell death due to serum-free conditions. Histograms represent wound closure 6 h after the scratch. Bars represent the means ± SEM of n = 6 of three independent experiments (N = 3). Differences between conditions were tested using the Wilcoxon signed rank test that compared each TKI condition *versus* control. *p < 0.05.

As endothelial cell migration is essential for wound healing and tissue regeneration, the ability of endothelial cells to migrate after treatment with the TKIs was evaluated by scratch assays. Assays were first performed in media containing 1% FBS to minimize the effects of cell proliferation on wound closure. In these conditions, there was no statistically significant difference between HUVECs treated with a TKI compared to control (**Supplementary Figure 4**). The absence of effect was possibly due to the high variability of the measurements due to the fact that scratch closure was slow with reduced cell proliferation. When we repeated the assay in media containing 10% FBS, dasatinib, even at infra-therapeutic concentration (0.005 µM) and bosutinib at high-dose (2 µM) inhibited scratch closure, whereas nilotinib facilitated wound healing (**Figure 5** and **Supplementary Figure 5**).

### Adhesion Molecule Expression

**Figure 6:**
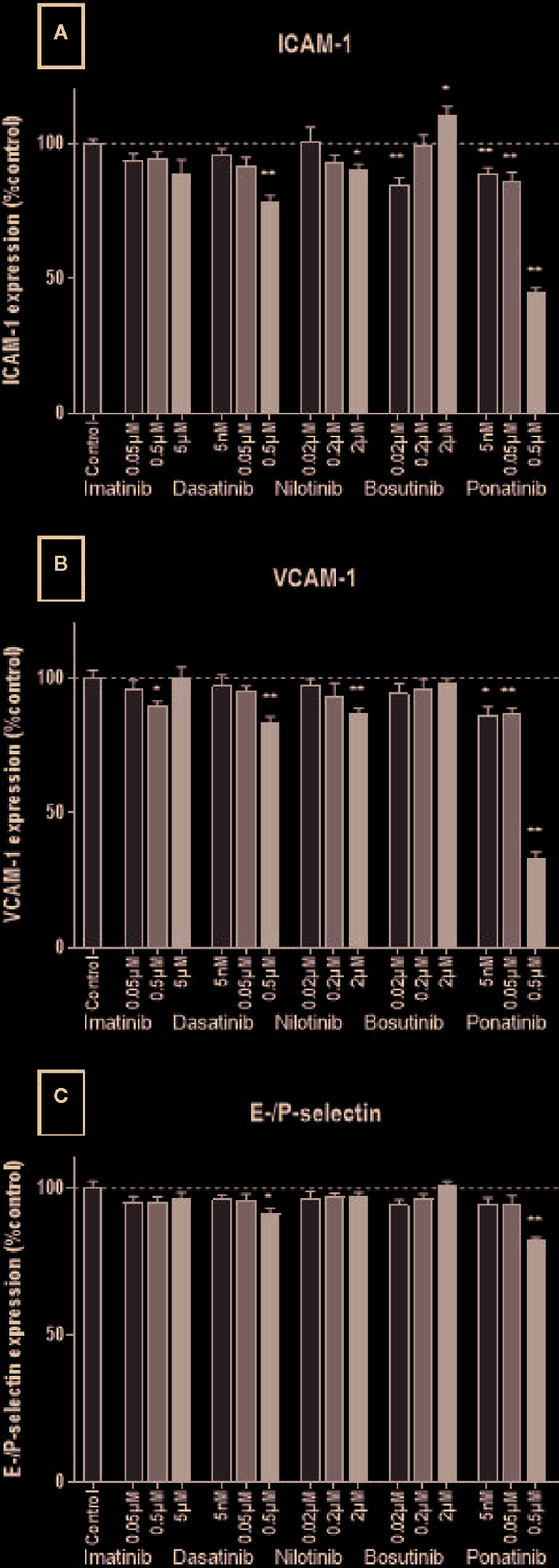
BCR-ABL TKIs do not increase adhesion molecule expression on HUVECs. Expression of ICAM-1 **(A)**, VCAM-1 **(B)**, and E-selectin and P-selectin **(C)** by HUVECs after a 4-h activation by 10 ng/ml of TNF-α followed by a 24 h treatment with BCR-ABL TKI in medium without FBS. Data are presented as means of the absorbance ± SEM of n = 9 of three independent experiments (N = 3). Three concentrations were tested for each TKI. Results are expressed relative to control (DMSO 0.2%). Differences between conditions were tested using the Wilcoxon signed rank test. *p < 0.05 and **p < 0.01.

Leukocyte recruitment is an important process in atherogenesis (Galkina and Ley, 2007). It requires the expression of adhesion molecules by activated endothelial cells. Here, we assessed in HUVECs the expression of ICAM-1, VCAM-1, and E-/P-selectin, three adhesion molecules involved in monocyte/macrophage recruitment (Čejková et al., 2016). Decreased expression of all three adhesion molecules was observed in HUVECs treated with dasatinib, nilotinib, and ponatinib at high concentrations for 24 h (0.5, 2, and 0.5 µM, respectively; **Figure 6**). Imatinib and bosutinib had no or little impact on adhesion molecule expression.

## Discussion

Our study demonstrates that BCR-ABL TKIs impact endothelial cells differently *in vitro* (**Table 1**). Ponatinib is the most cytotoxic for endothelial cells and induces apoptosis and necrosis, whereas the others BCR-ABL TKIs inhibited HUVEC proliferation. In addition, dasatinib and bosutinib delayed wound closure. These findings correlate with their clinical vascular safety profile (Aghel et al., 2017), and could explain why ponatinib is the BCR-ABL TKI that induces arterial occlusion the most frequently.

To our knowledge, the present study is the first to report that dasatinib affects endothelial cells from a different origin than pulmonary, indicating that the effect of dasatinib is not peculiar to pulmonary endothelial cells (Guignabert et al., 2016). Dasatinib inhibited endothelial cell proliferation at all the doses that we tested, and delayed wound closure. The comparison of the results of the scratch assay in the presence of 1% *versus* 10% FBS supports the hypothesis that delayed wound closure is the consequence of an inhibition of cell proliferation rather than an impact on endothelial cell migration. However, prior experiments suggested that dasatinib inhibits cell migration by altering the organization of the actin cytoskeleton and by inhibiting the formation of intercellular contacts (Dasgupta et al., 2017; Kreutzman et al., 2017). This impact on the endothelial cell cytoskeleton occurs through a disruption of the inhibitory signals from integrins to RhoA, a pathway implicating numerous tyrosine kinases, leading to RhoA/ROCK pathway activation. However, implicated tyrosine kinase(s) has/have not yet been identified. Interestingly, dasatinib did not increase ROS levels in endothelial cells, restraining the number of pathways by which dasatinib affects endothelial cell viability. In addition to its effects on endothelial cell proliferation and migration, dasatinib increased LDH release after 72 h of treatment, without inducing necrosis. This suggests that dasatinib does not induce endothelial cell death, but potentially induces cell damage. Interestingly, dasatinib protected cells against apoptosis and increased their metabolism, in accordance with the literature (Gover-Proaktor et al., 2018). This could explain why dasatinib is associated with a lower rate of arterial thrombosis than nilotinib and ponatinib.

Our results confirm the toxicity of ponatinib toward endothelial cells, endorsing the hypothesis that ponatinib would facilitate atherosclerosis and arterial thrombosis through alteration of endothelial cell viability (Katgi et al., 2015; Gover-Proaktor et al., 2016; Hadzijusufovic et al., 2017; Gover-Proaktor et al., 2018). Indeed, cell death within the arterial wall has already been recognized in atherosclerosis. Our study further provides insights about the mechanisms by which ponatinib dose-dependently affects endothelial cell viability. Ponatinib induced necrosis without increasing ROS levels, and might participate in atherosclerosis by this way (Paez-Mayorga et al., 2018). The identification of the type of endothelial cell death induced by ponatinib supports at least two hypotheses regarding the molecular signaling pathways responsible for this effect. One hypothesis would link necrotic cell death to a blockade of the cell cycle by the TKIs during progression from G1 to S phase. Interestingly, previous *in vitro* investigation reported a cell cycle arrest in the G1 phase in liver cancer cells treated with ponatinib, and identified that this cell cycle blockade was mediated by a reduction in the function of the CDK4/CDK6/Cyclin D1 complex (Liu et al., 2019). This complex is regulated by the prosurvival PI3K/Akt pathway, known to be impacted by some BCR-ABL TKIs, including ponatinib (Talbert et al., 2015). Therefore, future researches should analyze the effects of BCR-ABL TKIs on CDK4, CDK6, and Cyclin D1 levels/activities, and identify the implicated signaling pathways. A second hypothesis would link ponatinib-induced cell necrosis to Akt inhibition. Indeed, Akt is also highly implicated in the regulation of apoptosis signaling and mediates the responses of a majority of growth factors (Somanath et al., 2006), and ponatinib is known to inhibit Akt in cardiomyocytes (Singh et al., 2019). These observations highlight the need of a further thorough evaluation of this pathways in the endothelial response to ponatinib.

Interestingly, nilotinib had distinct impact on HUVECs. It increased LDH release after 72 h, but did not induce necrosis nor apoptosis at the tested concentrations, suggesting induction of non-fatal cell damage. It decreased HUVEC proliferation, but without affecting wound closure. These results are in line with the literature, and suggest a different etiology of vascular events. Other mechanisms than endothelial dysfunction with nilotinib should be explored. Because of its association with the development of hyperglycemia and hypercholesterolemia, accelerated atherosclerosis has been proposed as a potential mechanism of vascular thrombosis (Rea et al., 2014; Damrongwatanasuk and Fradley, 2017). However, it is currently unknown if the induction of these metabolic disorders alone is sufficient to explain the vascular complications associated with nilotinib. Another hypothesis is the induction of coronary artery spasms (Fiets et al., 2018).

Our work confirmed the little impact of imatinib on endothelial cells, in accordance with the literature (Vrekoussis et al., 2006; Hacker et al., 2007; Venalis et al., 2009; Gover-Proaktor et al., 2016; Guignabert et al., 2016; Sukegawa et al., 2017). Even if imatinib did not induce apoptosis nor necrosis, it increased the release of LDH after 72 h of treatment, suggesting the induction of non-fatal cell damage, similarly than with dasatinib and nilotinib. Bosutinib had little impact on endothelial cells. Yet, similarly to imatinib, it induced LDH release without inducing cell death, also suggesting the induction of cell damage. Interestingly, at high concentration (2 µM), it presented a profile close to that of dasatinib, as it inhibited cell proliferation, retarded wound closure and decreased apoptosis. Understanding the molecular pathways involved in endothelial cell death with therapy is important to develop more specific therapies. To anticipate the occurrence of such events and bring sufficient care to the vascular safety of new TKIs in clinical trials, *in vitro* endothelial testing of new drugs should be a requirement. Non clinical testing should include *in vitro* methods for assessing cell death, because global assays (such as the MTS and the LDH assays) may generate false-positive results and are not able to discriminate between cytotoxic and antiproliferative effects (Mery et al., 2017). The dose-dependent response seen here in most of the tests also suggests that the choice of the dose is important for *in vitro* experiments. This is one of the parameters that varied the most between studies, explaining most of the discrepancies between them. Of further note, numerous studies evaluated BCR-ABL TKIs *in vitro* using non-clinically relevant concentrations, thus leading to results that are not pertinent to clinical settings. One example is the report by Hadzijusufovic et al. that showed increased ICAM-1, VCAM-1 and E-selectin expression in HUVECs upon a 7.5 µM nilotinib treatment (Hadzijusufovic et al., 2017), whereas doses of nilotinib from 0.02 µM to 2 µM reduced the expression of the same protein (this study). Another important parameter that should be considered when designing the testing of compounds on endothelial functions *in vitro* is their binding to serum proteins, which would directly affect their biodisponibility.

## Perspectives and Conclusions

This study demonstrates that the responses of endothelial cells are different according to the TKI, corroborating the difference of their clinical vascular safety profiles. There are limitations to our study in that the findings are limited to one cell line (HUVECs). Even if this cell type is a good model for atherosclerotic studies (Onat et al., 2011), key observations should be confirmed on endothelial cells from other origin (*e.g.*, HCAEC) and using more elaborated models (*e.g.*, models involving vascular functions). BCR-ABL TKIs should also be tested on other cell lines than endothelial cells to assess if they would modulate other physiological functions. In addition, different time of TKI treatment should be tested, particularly for the assessment of adhesion molecule expression as Hadzijusufovic et al. reported increased in ICAM-1 and VCAM-1 cell surface levels after 4 h of nilotinib treatment (Hadzijusufovic et al., 2017).

To ascertain the relevance of these findings to human, it will be interesting to evaluate endothelial biomarkers in patients treated with BCR-ABL TKIs and to address their predictability. Circulating endothelial cells, endothelial progenitor cells and endothelial microparticles are good candidate biomarkers. They are measurable by blood sampling and have already been associated with damaged endothelium, as well as to predict the outcome of ischemic vascular diseases (Sabatier et al., 2009; Thomson and Garbuzova-Davis, 2016). In this context, Gover-Proaktor et al. suggested that endothelial progenitor cells may be more sensitive to ponatinib than mature endothelial cells, possibly because of a their stronger dependence on prosurvival factors and/or on the different penetration of ponatinib within the cells (Gover-Proaktor et al., 2016).

Our *in vitro-*based study analyzed the effects of BCR-ABL TKIs on endothelial cells with a special focus on cell death. It reports various effects of BCR-ABL TKIs on endothelial cells, suggesting a possible multifactorial cause and the possible implication of several off-targets in the development of cardiovascular events. Future researches should focus on the study of the molecular mechanisms that are responsible for endothelial cell death or impaired cell proliferation with BCR-ABL TKIs. Among the five commercialized BCR-ABL TKIs, ponatinib showed the most effects on endothelial cells. It reduced endothelial cell viability by inducing apoptosis and necrosis, which possibly facilitates the development of atherosclerosis through impaired endothelium permeability, enabling cell migration and the trapping of lipoproteins in the intima. Additionally, we found that dasatinib inhibits endothelial cell proliferation and can reduce endothelial cell migration, which might contribute to arterial thrombosis formation.

Because of the chronic use of these treatments, long-term safety is an important issue, and understanding the impact of these treatments may help in the design of new therapies. It may be of interest in the conception of strategies aiming at minimizing the risk of adverse events, *i.e.*, by avoiding the use of certain therapies in patients with pre-existing impairments in pathways disturbed by these therapeutic agents.

## Data Availability Statement

All datasets generated for this study are included in the article/**Supplementary Material**.

## Author Contributions

HH and JD were responsible for the conception and design of the study. HH and EM were responsible for the acquisition, analysis and interpretation of data. HH was responsible for drafting the manuscript. CB, A-SD, AW, PS, and J-MD contributed to the final draft of the manuscript. All authors agree to be accountable for the content of the work.

## Funding

This investigation study was not funded by any organizations or pharmaceutical companies. PS is a Senior Research Scientist of the Belgian National Fund for Scientific Research (F.R.S.-FNRS).

## Conflict of Interest

JD is CEO of QUALIblood s.a. He reports fees from Bioxodes, Mithra Pharmaceuticals, Daiichi-Sankyo, DOASense, Diagnostica Stago, Portola Pharmaceuticals, Roche, Roche Diagnostics, all unrelated to the present work.

The remaining authors declare that the research was conducted in the absence of any commercial or financial relationships that could be construed as a potential conflict of interest.
