# Supplementary material for: The Risk of Arterial Thrombosis in Patients With Chronic Myeloid Leukemia Treated With Second and Third Generation BCR-ABL Tyrosine Kinase Inhibitors May Be Explained by Their Impact on Endothelial Cells: An In-Vitro Study
Source: Front Pharmacol. 2020 Jul 3;11:1007. doi: 10.3389/fphar.2020.01007 (PMC7350860; doi:10.3389/fphar.2020.01007)
Supplement: Supplementary file 1 [file DataSheet_1.docx]

Supplementary Material

# eFigure 1: Ponatinib affects endothelial cell viability after 24 h.

MTS (**A**) and LDH (**B**) assays were performed on HUVECs exposed for 24 h to the indicated BCR-ABL TKI in medium with 10% dialyzed FBS. Data are presented as means ± SEM of n = 9 of three independent experiments (N = 3). Results are expressed relative to control (DMSO 0.2%). Differences between conditions were tested using the Wilcoxon signed rank test that compared the effect of each TKI condition *versus* control. *p < 0.05 and **p < 0.01.

# eFigure 2: BCR-ABL TKIs did not increase HUVEC apoptosis or late-apoptosis/necrosis after 24 h.

HUVECs were labeled with Annexin V^FITC^ and 7-AAD after exposure to BCR-ABL TKIs for 24 h in medium with 10% dialyzed FBS. The percentage of Annexin V^+^/7-AAD^-^ and 7-AAD^+^ cells revealed early apoptotic (**A**) and late-apoptotic/necrotic (**B**) HUVECs, respectively. Bars represent the means of the three experiments ± SEM. Results are expressed relative to control (DMSO 0.2%). Differences between conditions were tested using a one sample t-test that compared each TKI condition *versus* control.

# eFigure 3: Imatinib, nilotinib and bosutinib inhibit HUVEC proliferation after 72 h.

Cell cycle analysis was performed on HUVECs exposed to BCR-ABL TKI for 72 h in medium with 10% dialyzed FBS by measuring EdU incorporation and DNA content (FxCycle). Histograms represents cells in S-phase (**A**), G0/G1 phase (**B**) and G2/M phase (**C**). Bars represent the means of three experiments ± SEM. Three concentrations were tested for each TKI. Results are expressed relative to control (DMSO 0.2%). Differences between conditions were tested using a one sample t-test that compare each TKI condition *versus* control. *p<0.05 and **p<0.01.

# eFigure 4: BCR-ABL TKIs do not affect scratch closure in media containing 1% FBS.

Endothelial cell migration was assessed by a scratch assay after exposure to BCR-ABL TKIs during 24 h. The scratch assay was performed in 1% FBS media to minimize the impact of cell proliferation. The histogram represents wound closure 6 h after the scratch. Bars represent the means ± SEM of n = 6 of three independent experiments (N = 3). Differences between conditions were tested using the Wilcoxon signed rank test that compared each TKI condition *versus* control.

**eFigure 5:** **Dasatinib and bosutinib inhibited wound closure.**


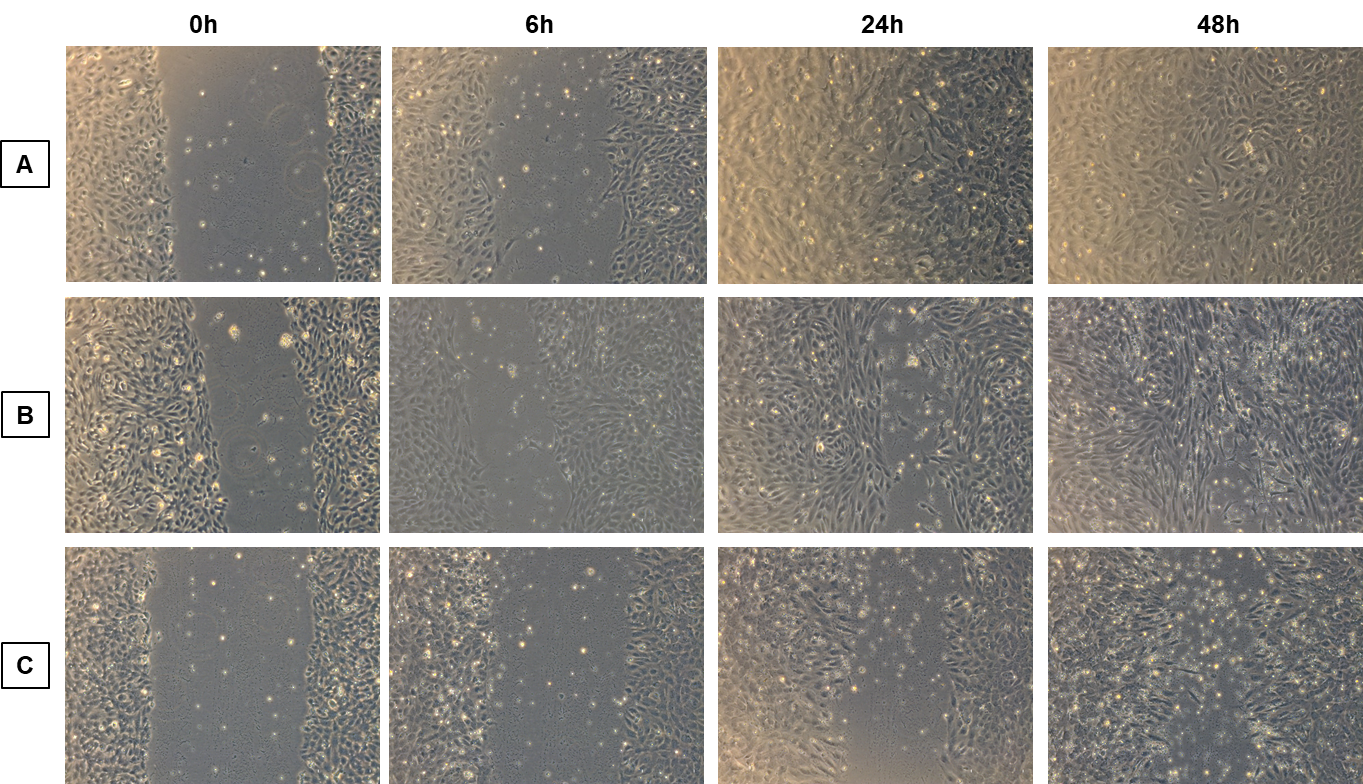
Endothelial cell migration was assessed by a scratch assay after exposure to BCR-ABL TKIs during 24 h. The scratch assay was performed in 10% FBS media. Pictures are representative images of scratch closure with control (0.2% DMSO) (**A**), dasatinib 0.5µM (**B**) and bosutinib 2µM (**C**) at the time of the scratch (0h), after 6h, 24h and 48h.
